# Self-reported energy intake of male & female bodybuilders in the scientific literature

**DOI:** 10.1186/1550-2783-9-S1-P19

**Published:** 2012-11-19

**Authors:** Brad Lankford, Bill I Campbell

**Affiliations:** 1 University of South Florida, Tampa, FL, 33620, USA

## Background

Nutrient intake is critical to a bodybuilder in terms of improving the overall muscular appearance of their physique. Total energy intake and the proportion of the kilocalories derived from carbohydrates, protein, and fats are often precisely planned and implemented to maximize skeletal muscle hypertrophy and reduce body fat. The purpose of this study is to describe the self-reported energy intakes of male and female bodybuilders and to determine if differences exist between the genders in regards to total energy intake and macronutrient composition.

## Methods

A comprehensive literature review was performed using the PubMed database. Every effort was made to generate all relative articles pertaining to male and female bodybuilders’ self-reported energy intakes. The search yielded a total of 13 articles, 8 male bodybuilder studies and 5 female bodybuilder studies. The studies summarized contained professional, collegiate, and international bodybuilders during the offseason or non-competitive/non-dieting phase. In 12 of the 13 studies included, energy intakes were derived from food records ranging from 3 days to 7 days. The other study used a food frequency questionnaire. Total kilocalories, kilocalories/kg of body mass, kilocalories/kg of fat-free mass (FFM) and macronutrient composition were recorded and analyzed. Differences between male and female bodybuilders were analyzed via an independent samples t-test using IBM SPSS Statistics (v20).

## Results

All data are reported as means ± standard deviations. Total kilocalories were 4,049 ± 892 and 2,067 ± 525 for male and female bodybuilders, respectively. The males ingested significantly more total kilocalories than the females (p = 0.001). When kilocalories were expressed per kilogram of body weight, male bodybuilders ingested 47.4 ± 10 and females ingested 35.8 ± 9. No significant differences existed between male and female bodybuilders (p = .064). When kilocalories were expressed per kilogram of FFM, male bodybuilders ingested 54.3 ± 12 and female bodybuilders ingested 41.6 ± 11. There were no significance differences in the amount of kilocalories per kilogram of FFM (p = .126). Total % of carbohydrate ingested was 48 ± 6% and 54 ± 3% for males and females, respectively. No significant differences were demonstrated between the genders (p = .070). The total % of protein ingested for males were 21 ± 2% and females was 24 ± 6%. No significant differences were demonstrated (p = .245). The total % of fat ingested for males were 31 ± 4% and females was 25 ± 8%. Although males reported a higher percentage of total fat ingested, no significant differences existed (p = .060).

## Conclusions

Based on the data, male bodybuilders reported ingesting significantly more total kilocalories than female bodybuilders. However, when adjusted for body mass and fat free mass, no significant differences exist between the genders. In relation to macronutrient composition (% Carbohydrate, % Protein, & % Fat), no significant differences exist between male and female bodybuilders.

